# Pannexin 1 drives efficient epithelial repair after tissue injury

**DOI:** 10.1126/sciimmunol.abm4032

**Published:** 2022-05-13

**Authors:** Christopher D. Lucas, Christopher B. Medina, Finnius A. Bruton, David A. Dorward, Michael H. Raymond, Turan Tufan, J. Iker Etchegaray, Brady Barron, Magdalena E.M. Oremek, Sanja Arandjelovic, Emily Farber, Suna Onngut-Gumuscu, Eugene Ke, Moira KB Whyte, Adriano G. Rossi, Kodi S. Ravichandran

**Affiliations:** 1 Center for Cell Clearance, Department of Microbiology, Immunology and Cancer Biology, University of Virginia, Charlottesville, VA, USA; 2 University of Edinburgh Centre for Inflammation Research, Queen's Medical Research Institute, Edinburgh BioQuarter, UK; 3 Institute for Regeneration and Repair, Edinburgh BioQuarter, UK; 4 Center for Public Health Genomics, University of Virginia, Charlottesville, VA, USA; 5 Department of Microbiology, Immunology and Cancer Biology, University of Virginia, Charlottesville, VA, USA; 6 Inflammation Research Centre, VIB, and the Department of Biomedical Molecular Biology, Ghent University, Ghent, Belgium; 7 Division of Immunobiology, Department of Pathology and Immunology, Washington University School of Medicine, St. Louis, MO, USA

## Abstract

Epithelial tissues such as lung and skin are exposed to the environment and therefore particularly vulnerable to damage during injury or infection. Rapid repair is therefore essential to restore function and organ homeostasis. Dysregulated epithelial tissue repair occurs in several human disease states, yet how individual cell types communicate and interact to coordinate tissue regeneration is incompletely understood. Here, we show that pannexin 1 (Panx1), a cell membrane channel activated by caspases in dying cells, drives efficient epithelial regeneration after tissue injury by regulating injury-induced epithelial proliferation. Lung airway epithelial injury promotes the Panx1-dependent release of factors including ATP, from dying epithelial cells which regulates macrophage phenotype post-injury. This process, in turn, induces a reparative response in tissue macrophages that includes the induction of the soluble mitogen amphiregulin, which promotes injury-induced epithelial proliferation. Analysis of regenerating lung epithelium identified Panx1-dependent induction of *Nras* and *Bcas2,* both of which positively promoted epithelial proliferation and tissue regeneration *in vivo.* We also established that this role of Panx1 in boosting epithelial repair after injury is conserved between mouse lung and zebrafish tailfin. These data identify a Panx1-mediated communication circuit between epithelial cells and macrophages as a key step in promoting epithelial regeneration post-injury.

## Introduction

The ability to repair lost and injured tissue is of critical importance to maintain organ function and the survival of the organism (1, 2). Epithelial tissues are often the primary targets of tissue damage given their frequent exposure to the external environment. This is particularly relevant within the lungs where the necessity for gas exchange brings in thousands of litres of air from the external environment every day. Indeed, lung epithelial injury and cell death are central to the pathogenesis of both chronic lung disease (such as asthma, chronic obstructive pulmonary disease, and lung fibrosis) and acute pulmonary insults (including bacterial pneumonia, viral infections and inhalation of toxins) (3). The consequences of widespread lung epithelial injury have been particularly evident during the global SARS-CoV-2 pandemic, where overwhelming pulmonary epithelial injury and aberrant repair leads to respiratory failure and substantial mortality in severe cases (4, 5). Understanding endogenous processes that drive swift and coordinated tissue repair are therefore of high importance, especially given that no existing treatments specifically target epithelial repair.

The immune response to tissue injury has a central role in dictating the functional outcome, with most evidence suggesting that macrophage lineage cells acting as positive mediators of tissue repair. Macrophage recruitment and expansion have been reported to mediate successful repair in response to diverse tissue insults and this response is evolutionarily conserved, as evidenced by limb amputation in axolotl, tailfin transection in zebrafish, experimental influenza lung infection, and aberrant wound healing/organ fibrosis (6–9). Furthermore, substantial macrophage expansion is seen in fatal human lung injury (4). However, the mechanisms by which macrophages engage in bidirectional crosstalk with epithelial cells to sense wounding and control tissue repair, as well as the identity of local environmental cues that enhance macrophage delivery of 'pro-reparative' signals, remain poorly understood.

Cell death occurs during tissue homeostasis and disease, with large numbers of dead and dying cells induced during tissue injury and inflammation (10, 11). Apoptotic cells are well-recognised to have pleiotropic anti-inflammatory actions, but evidence also exists for them having a role in tissue regeneration (12). For example, in injured Hydra, apoptotic cells are both necessary and sufficient to promote head regeneration (13). Much attention has focused on apoptotic cell interactions with surrounding phagocytes, especially the 'eat me' signals that promote engulfment by nearby cells including macrophages (11). However, dead and dying cells can also actively direct tissue and cellular programs independently of engulfment by the release of soluble mediators, including metabolites released during apoptosis. One source of metabolite release is via caspase-mediated opening of pannexin 1 (Panx1) channels at the cell membrane of cells undergoing apoptosis (14, 15). Panx1 is a heptameric plasma membrane channel that is activated by caspases during cell death due to cleavage of a specific C-terminal site. This was originally identified as a mediator of the find-me signal/nucleotide release from apoptotic cells in the context of recruiting macrophages to apoptotic cells (16, 17). Recent students suggest that metabolites released via Panx1 from apoptotic cells can be part of the communication of dying cells with the surrounding microenvironment, with implications for dampening tissue inflammation (14).

This prompted us to ask whether dying lung epithelial cells communicate via Panx1 within the lung tissue microenvironment to influence tissue regeneration after acute epithelial injury. In the current work, we identify Panx1 channels as one of the core determinants of establishing an epithelial – macrophage circuit that promotes efficient injury-induced epithelial proliferation and tissue repair. Addressing this in the context of acute lung injury, we find that Panx1-dependent communication imprints an injury phenotype on local tissue macrophages that in turn enhances positive regulators of epithelial proliferation in the regenerating tissue.

## Results

### Pannexin 1 drives efficient epithelial repair after tissue injury

**Figure 1:**
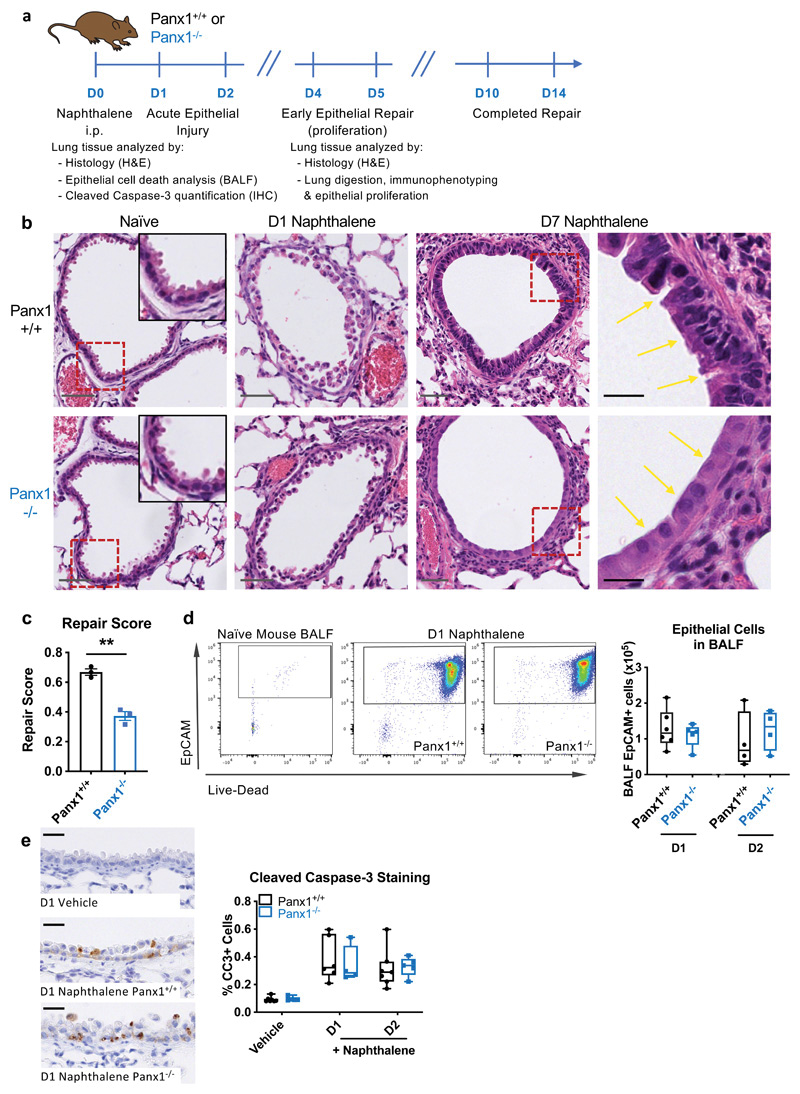
Pannexin1 is required for efficient epithelial repair after tissue injury. (a) Wild type (Panx1+^/^+) or pannexin 1 globally deficient mice (Panx1^-/-^) were injected with naphthalene (200mg/kg) i.p. to cause acute airway epithelial death prior to analysis of bronchoalveolar lavage fluid (BALF) or lung tissue. (b) Representative H&E staining of lung tissue sections from naïve Panx1+^/^+ or Panx1^-/-^ mice and at day 1 (D1) and D7 post-naphthalene injury, with injured and repairing airway epithelium highlighted by yellow arrows. Grey scale bars, 50μm; black scale bars, 20μm. Dotted red lines reflect insets in naïve images and zoomed in images in D7 naphthalene images. (c) H&E sections were blinded prior to analysis by a lung pathologist (n=3 per group, assessed by t-test, one independent experiment). (d) BALF was acquired at day 1 or day 2 post-naphthalene injury and cellular content analysed for epithelial cells (CD45^-^/EpCAM^+^ cells) and their staining with a live-dead (LD) marker (low staining of LD marker are live cells, high staining are dead cells; n=4-6, two independent experiments). (e) Caspase-3 activation was assessed by cleaved caspase-3 staining on lung tissue sections in the absence of injury and on days 1 and 2 after naphthalene epithelial injury (n=4-7, two independent experiments). Black scale bars, 20um. **p<0.01.

To better understand how epithelial cells and macrophages establish crosstalk to mediate tissue repair, we utilized a model of acute airway epithelial injury induced by naphthalene (18). Naphthalene administration leads to early airway epithelial cell death and caspase activation; this is followed by gross disruption of the normal airway epithelial architecture (days 1-2 after injury) and tissue repair over a period of 10-14 days (see Fig. 1a). Naphthalene-induced airway epithelial injury was studied, as airway epithelium is frequently the primary target of pulmonary insults, and airway epithelium expresses pannexin 1 (Panx1) protein at a high level within the lung (The Human Protein Atlas).

Based on recent studies that suggest that Panx1 channels release metabolites from apoptotic cells that help dampen tissue inflammation, we asked whether Panx1 might also play a role in epithelial repair after naphthalene-induced tissue injury. To address this, we subjected mice globally deficient in Panx1 (Panx1^-/-^) or littermate controls to naphthalene-induced airway epithelial injury and analysed the lungs via immunohistochemistry. Both Panx1^+/+^ and Panx1^-/-^ mice had similar levels of early airway epithelial injury, death, and gross morphological disruption (Fig. 1b), while maintaining alveolar architecture. However, at day 7 post-injury, there was persisting abnormal epithelial morphology in the Panx1^-/-^ mice (Fig. 1b,c). When comparing the two groups of mice in this model of injury, Panx1^-/-^ mice had features of 'simple' low cuboidal airway epithelium rather than the typical columnar morphology, accompanied by failure to re-establish the abundant 'hobnail' eosinophilic cytoplasm and a fine nuclear chromatin pattern (Fig. 1b). The Panx1^-/-^ mice also had fewer nuclei within the epithelium of airways, with clear spacing between adjacent cells rather than the hyperplastic, overlapping of nuclei seen at the same time point in the control mice, consistent with a low cellular density of the epithelium (Fig. 1b,c). Together, these data suggested defective epithelial regeneration when Panx1 expression is disrupted.

The morphological features noted above could have arisen either by Panx1^-/-^ mice experiencing initial greater injury with more acute epithelial cell loss or defective regeneration of new epithelial cells in response to injury. To test these possibilities, we quantified the exfoliated dead epithelial cells recovered in bronchoalveolar lavage fluid (BALF) after induction of naphthalene-induced tissue injury. Large numbers of epithelial cells (CD45^-^ EpCAM^+^ cells) that almost uniformly stained positive with a live-dead marker were recovered, yet the numbers of dead epithelial cells were similar between Panx1+^/^+ and Panx1^-/-^ mice (Fig. 1d, S1). In addition, when we quantified cleaved caspase-3 staining of lung tissue sections, the increased caspase-3 cleavage observed after naphthalene injury was similar in Panx1+^/^+ and Panx1^-/-^ mice (Fig. 1e). These data suggested that Panx1 does not influence the initial level of cell death/ or tissue injury, and that a defect in tissue repair was more likely.

**Figure 2:**
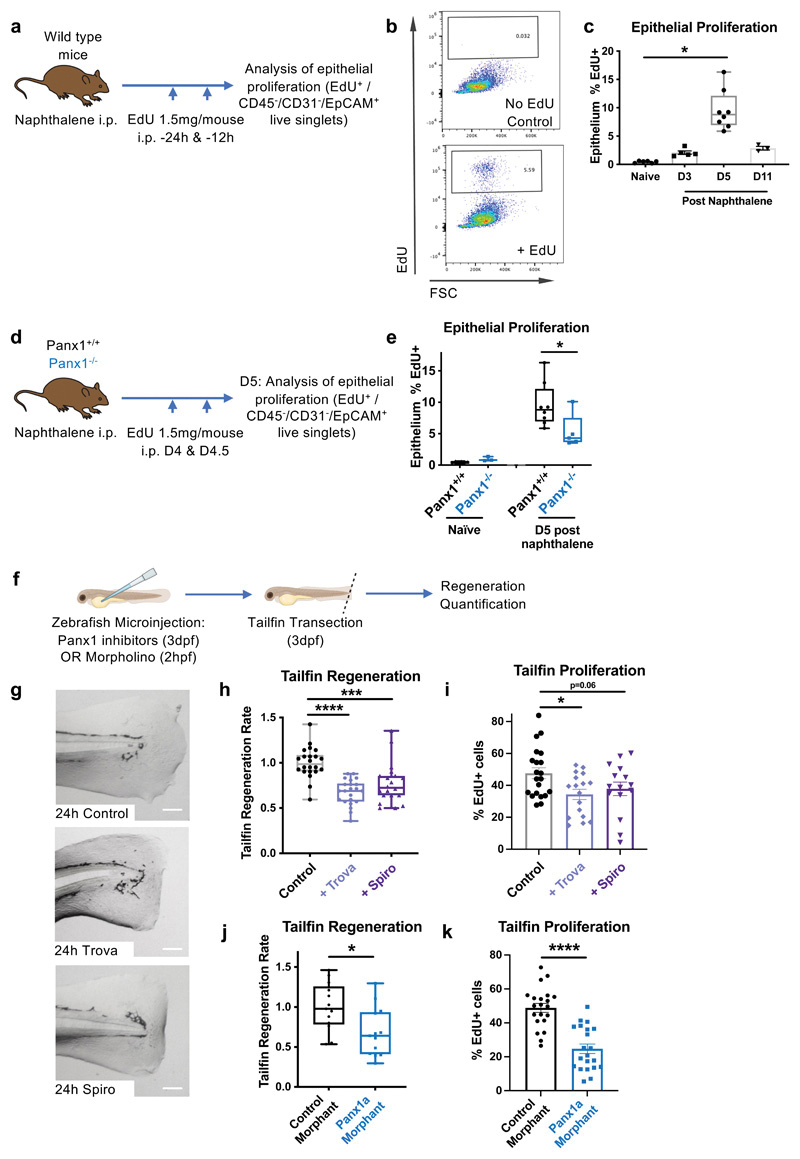
Pannexin1 promotes injury-induced epithelial proliferation, and boosts tailfin regeneration in zebrafish. (a-c) *In vivo* epithelial injury-induced proliferation in response to naphthalene was analysed by EdU incorporation of CD45^-^/CD31^-^/EpCAM^+^ cells (n=3-8, analysed by one-way ANOVA with Dunnett's multiple comparisons test). (d,e) Injury-induced epithelial proliferation was measured in Panx1+^/^+ and Panx1^-/-^ mice, with naïve mouse data shown for comparison (n=3-6 naïve, n=5-8 post-naphthalene, two independent experiments, assessed by t-test). (f) Zebrafish at 3 days post-fertilisation were microinjected with 1nL of the Panx1 pharmacological inhibitors trovafloxacin (80mM) or spironolactone (120mM), or DMSO control, and tailfins were transected. (g,h) Tailfin regeneration rate was assessed at 48h (n=20-22 separate animals, three separate experiments, assessed by one-way ANOVA with Holm-Šídák's multiple comparisons test). Scale bar, 100um. (i) Proliferation in the regenerating tailfin analysed by EdU incorporation, imaged by light sheet fluorescent microscopy (n=15-21 per group, assessed by Holm-Šídák's multiple comparisons test). (j) Similarly, Panx1a morphants (or control sequence morphants) underwent tailfin transection and regeneration rate was assessed at 48h post-injury (n=13 separate animals, two separate experiments, assessed by t-test), with (k) tailfin proliferation analysed by EdU incorporation, (n=21 per group, one experiment, assessed by t-test). *p<0.05, ***p<0.001, **** p<0.0001.

Proliferation of the remaining live cells to replace those that were lost during injury is an important component of tissue repair. Therefore, we asked whether altered epithelial proliferation contributes to the abnormal epithelial repair observed in Panx1^-/-^ mice. We injected mice with EdU, which is incorporated into DNA of proliferating cells, and performed flow cytometric analysis of EdU incorporation by lung epithelial cells (CD45^-^/CD31^-^/EpCAM^+^ cells) (Fig.2a,b). This confirmed that early epithelial repair (the usual peak of epithelial proliferation) occurred in day 4-5 post-injury (Fig.2c). In the Panx1^-/-^ mice, we observed a significant reduction in injury-induced proliferation during the usual peak of epithelial proliferation (Fig. 2d,e).

To test whether Panx1 was more broadly involved in epithelial repair, we used a second model of acute epithelial injury and repair - the regeneration of the resected tailfin in the zebrafish. In this model, surgical tailfin transection is performed at 3 days post-fertilisation, and spontaneous tailfin regeneration is measured serially in individual fish over the next 48h (Fig. 2f). We tested the involvement of Panx1 in this model via pharmacological and genetic approaches. Zebrafish pre-treated with the small molecule Panx1 inhibitors trovafloxacin or spironolactone (19, 20) had diminished tailfin regeneration (Fig. 2g,h) as well as impaired proliferation in the regenerating tailfin (analysed by EdU incorporation; Fig. 2i). Genetically, targeting of the Panx1 homologue in zebrafish *(Panx1a)* via morpholino (21) showed similarly diminished tailfin regeneration (Fig 2j) and proliferation (Fig 2k). Taken together, these data demonstrated that Panx1 positively regulates tissue repair in response to diverse epithelial insults, at least in part, by augmenting injury-induced epithelial proliferation, and that the function of Panx1 in tissue repair is evolutionarily conserved.

### Macrophages expand and augment epithelial proliferation after tissue injury

**Figure 3:**
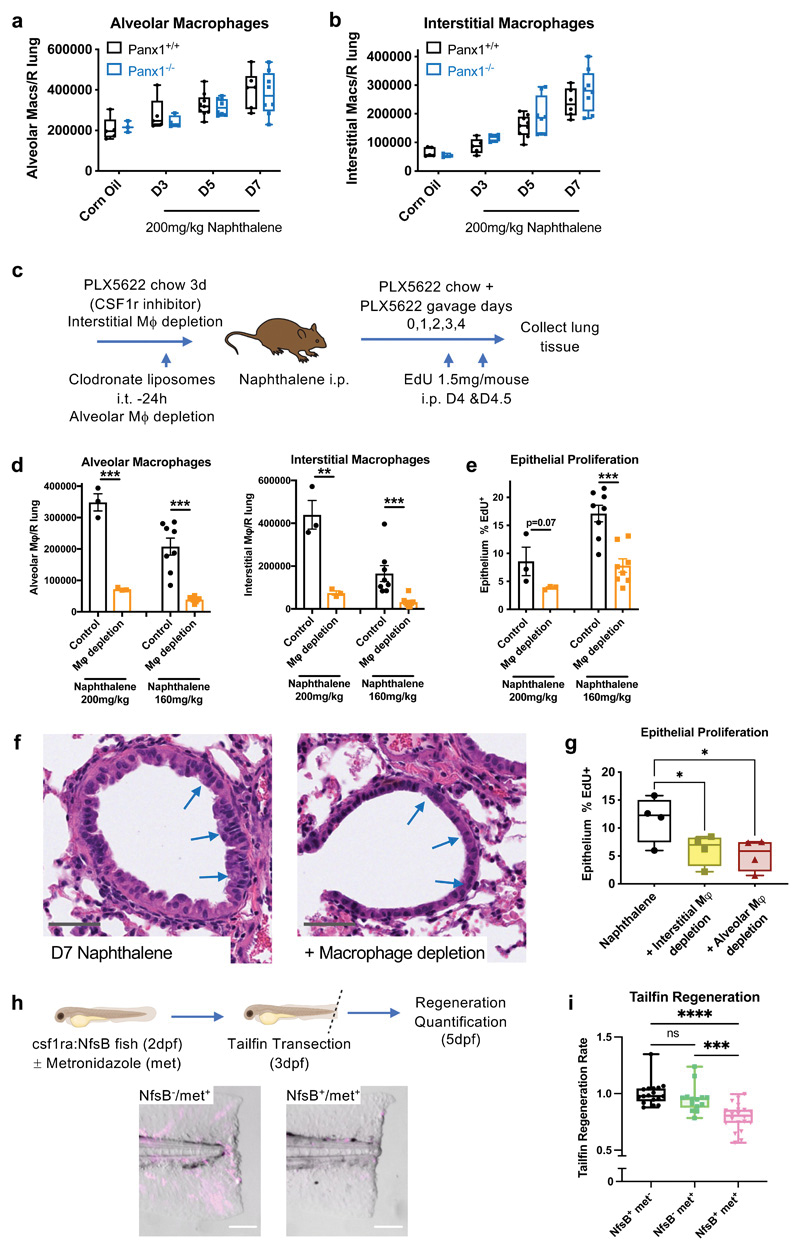
Macrophages expand and augment epithelial proliferation in response to tissue injury. (a) Lung alveolar macrophage numbers (CD45^+^/CD11c^+^/Siglec-F^+^ cells) and (b) interstitial macrophage numbers (CD45^+^/CD11b^+^/CD64^+^ cells) in digested right lung lobes after naphthalene-induced epithelial injury (200mg/kg) analysed by flow cytometry of lung digests in Panx1+^/^+ and Panx1^-/-^ mice (n=3-5 corn oil; n=5-9 post-naphthalene). (c) Schema of experimental protocol for lung macrophage depletion in Panx1+^/^+ mice, achieved by intratracheal (i.t.) administration of 100μL clodronate liposomes to deplete alveolar macrophages, and PLX5622 containing chow (1200mg/kg feed) ad libitum for 3d prior to experimentation with additional PLX5622 (65mg/kg) given by oral gavage (on days 0,1,2,3&4 of naphthalene administration) to deplete interstitial macrophages. (d) Confirmation of macrophage depletion after epithelial injury and (e) *In vivo* epithelial injury-induced proliferation analysed by EdU incorporation (EdU 1.5mg/mouse given intraperitoneally (i.p.) on days 4&4.5) with or without macrophage depletion in response to high dose (200mg/kg, n=3) or intermediate dose (160mg/kg n=8) naphthalene, assessed by t-test. (f) Representative H&E staining of lung tissue sections at day 7 after naphthalene injury (200mg/kg) with or without macrophage depletion, with injured and repairing airway epithelium highlighted by blue arrows. Grey scale bars, 50um. (g) *In vivo* epithelial injury-induced proliferation analysed by EdU incorporation with alveolar macrophage depletion (by clodronate liposomes) or interstitial macrophage depletion (by PLX5622) in response to naphthalene (160mg/kg, n=4/group, one experiment, assessed by one-way ANOVA with Holm-Šídák's multiple comparisons test). (h) Macrophage ablation achieved using the csf1ra:NfsB line (NfsB) and metronidazole (met) treatment (NfsB^+^/met^-^, NfsB^-^/met^+^ and NfsB^+^/met^+^ groups; macrophage depletion only with NfsB^+^/met^+^) with macrophage ablation confirmed in NfsB^+^/met^+^ fish by fluorescent microscopy (mCherry^+^ macrophages pseudocoloured in magenta) scale bar 100um. (i) Defective tailfin regeneration in macrophage-deficient zebrafish (NfsB^+^/met^+^ group) (n=14, assessed by one-way ANOVA with Holm-Šídák's multiple comparisons test). *p<0.05, **p<0.01 *** p<0.001, ****p<0.0001.

To identify a mechanism by which Panx1 could regulate injury-induced epithelial proliferation and repair, we first examined the role of macrophages. We focused on macrophages as they have diverse roles in tissue injury and can influence cells that make up the structural environment of a given organ, including epithelial cells (2). Furthermore, local macrophage numbers frequently increase during tissue injury, including zebrafish tailfin transection and in fatal human lung injury (4, 10, 22). There are two major populations of lung macrophages - alveolar macrophages within the luminal aspect of airways and alveoli, and interstitial macrophages that exist within lung tissue; both of these populations are distinguishable by specific markers by flow cytometry and by gene expression profiles (23, 24) (Fig. S2). Detailed analysis of macrophage numbers within lung tissue in response to naphthalene-induced epithelial injury demonstrated that both macrophage populations expanded in the repair phase (days 5-7 post-injury). Interestingly, there was much greater expansion of interstitial macrophages compared to alveolar macrophages, with interstitial macrophage numbers increasing ˜3 fold post-injury and alveolar macrophages increasing ˜1.5 fold post-injury (Fig. 3a,b).

To test whether macrophages were essential for efficient epithelial proliferation and repair after airway injury, we concurrently depleted both alveolar and interstitial macrophages, as we did not know if there would be any functional redundancy between alveolar and interstitial populations within the context of airway injury and regeneration. We first optimized conditions for depleting alveolar macrophages using clodronate liposomes that were instilled via intra-tracheal administration (25). To deplete interstitial macrophages, we blocked the colony stimulating factor 1 receptor (CSF1r/CD115) via the CSF1r selective inhibitor PLX5622 (Fig. 3c, S3). These two distinct methods for achieving depletion of alveolar and interstitial macrophage populations also allowed temporal control of macrophage deficiency. After extensive optimisation to deplete both macrophage populations synchronously, we asked how macrophage depletion impacts epithelial repair after naphthalene-induced tissue injury. Targeting both alveolar and interstitial macrophage populations led to impaired epithelial injury-induced proliferation in response to both high dose and intermediate doses of naphthalene (Fig. 3c-f), confirming macrophages as positive regulators of injury-induced epithelial proliferation. Interestingly, selective depletion of either alveolar macrophages (with liposomal clodronate) or interstitial macrophages (with the CSF1r inhibitor PLX5622) was sufficient to attenuate injury-induced epithelial proliferation (Fig. 3g). Similarly, we also investigated whether the macrophage expansion at the wound site following tailfin injury (10) was functionally involved in tailfin regeneration. We utilized the transgenic csf1ra:NfsB zebrafish line [transgenic(csf1ra:gal4;UAS:mCherry-NfsB)], which targets the nitroreductase enzyme (NfsB) to macrophages, rendering them susceptible to metronidazole-induced ablation (Fig. 3h). Diminished tailfin regeneration following transection was observed in the macrophage-depleted zebrafish (NfsB^+^/met^+^ group), confirming macrophages as functionally important in tailfin regeneration and correlating with the role of Panx1 in this process (Fig. 3i).

### Epithelial cell pannexin 1 regulates injury-induced proliferation

**Figure 4:**
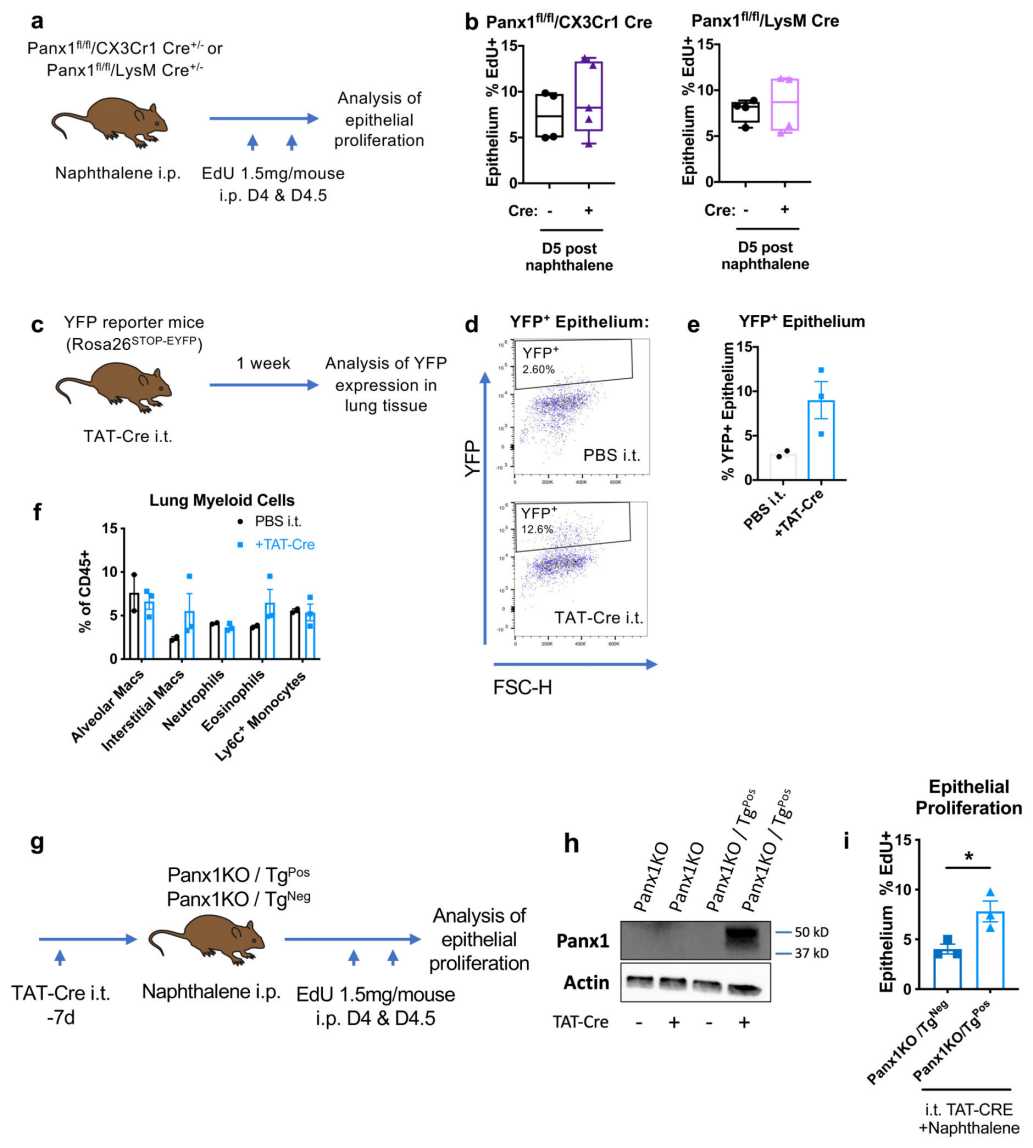
Pannexin1 at the site of tissue injury regulates epithelial injury-induced proliferation. (a) Schema of experimental protocol for deleting Panx1 using CX3Cr1-Cre or LysM-Cre mediated approaches prior to epithelial injury, with (b) *in vivo* epithelial injury-induced proliferation analysed by EdU incorporation (n=4-5/group Cx3Cr1 Cre, assessed by t-test; n=4/group LysM Cre, assessed by t-test). (c) Schema of local delivery of TAT-Cre into the lungs. (d) Representative flow cytometry plots of YFP expression within epithelium with either PBS (vehicle) or TAT-Cre delivery with (e) confirmation of TAT-Cre-dependent YFP expression within epithelium and (f) analysis of major immune cell populations in response to TAT-Cre. (g) Schema of experimental protocol for expressing Panx1 transgene (Tg) on a global Panx1-deficient mouse (Panx1^-/-^) using i.t. TAT-Cre administration. (h) Panx1 protein expression on whole lung extracts analysed in Panx1^-/-^/Tg^-^ and Panx1^-/-^/Tg+ mice with and without local TAT-Cre, confirming Panx1 expression in a Tg-dependent and Cre-dependent fashion. (i) *In vivo* epithelial injury-induced proliferation (analysed by EdU incorporation post-naphthalene) is boosted by re-expression of Panx1 at the site of tissue injury (n=3/group, one experiment, assessed by t-test) with both genotypes treated intra-tracheally with TAT-Cre. *p<0.05.

Our data thus far demonstrated that loss of Panx1 can impair epithelial injury-induced proliferation, and that depleting lung macrophages similarly limits epithelial proliferation after injury. We next asked whether Panx1 expression in macrophages was important for promoting epithelial proliferation after injury using two different genetic approaches. First, we crossed *Panx1^fl/fl^* mice to LysM-Cre mice, where Cre-mediated deletion occurs broadly within the myeloid population including macrophages, dendritic cells, and neutrophils (26, 27). As a second approach, we crossed *Panx1^fl/fl^* mice to *Cx3Cr1-Cre* mice to target macrophages (28), and we confirmed that both alveolar and interstitial macrophage populations expressed Cre with high efficiency (Fig. S4). We then subjected the *LysM-Cre Panx1^fl/fl^* or *Cx3Cr1-Cre Panx1^fl/fl^* mice to naphthalene-induced injury, and assessed the proliferation of the epithelium, using EdU incorporation as a readout (Fig. 4a). Neither *LysM-Cre-* or Cx3Cr1-Cre-mediated *Panx1* deletion altered injury-induced epithelial proliferation and repair (Fig. 4b), suggesting that macrophage expression of Panx1 is likely dispensable for efficient injury-induced epithelial proliferation.

To investigate whether epithelial cell expression of Panx1 was important, we utilized a recently generated Panx1 transgenic mouse line generated in our laboratory. This Panx1^flox-STOP-flox^ mouse line is capable of conditional expression of a human Panx1 transgene, where the transgene is preceded by a STOP cassette flanked by loxP sites that allows Cre-mediated expression of the transgene (28). To induce transgenic Panx1 expression, we developed an approach of local Cre delivery to the lung via TAT-Cre, which is a recombinant form of Cre fused to TAT sequence that allows entry of the Cre protein into the cells. To ensure that local administration of Cre by intratracheal delivery of TAT-Cre would be sufficient to tun on our transgene, we initially optimised this approach in a YFP reporter mouse strain (Fig. 4c).This confirmed that intratracheal delivery of Cre led to YFP expression within the epithelium (Fig. 4d,e). Importantly, the TAT-Cre administration itself induced minimal or undetectable inflammation (Fig. 4f). Next, to determine whether restoration of Panx1 could rescue the phenotype of the Panx1-null background, we crossed the Panx1^flox-STOP-flox^ transgenic mice to the global Panx1- deficient background (*Panx1KO/Tg*, Fig. 4g). We then induced Panx1 expression via TAT-Cre (Fig. 4h) and subjected these mice to naphthalene-induced injury. Remarkably, re-expression of Panx1 in epithelial and luminal cells of the lung was sufficient to boost injury-induced epithelial proliferation (Fig.4i), further demonstrating that Panx1 expression in lung epithelial cells is important for tissue repair.

### Pannexin 1 initiates an epithelium-macrophage repair circuit

Naphthalene-induced injury causes epithelial apoptosis with caspase-3 activation (Fig. 1e), and it has been demonstrated that caspase-dependent activation of pannexin channels during apoptosis leads to release of metabolites. Such metabolites then communicate with other live cells within the tissue neighbourhood and help to dampen inflammation in models of arthritis and lung transplantation (14). Therefore, we next tested a working model in which the release of soluble mediators from the apoptotic epithelial cells via pannexin could induce changes in lung macrophages, which in turn would produce factors that would help repair the injured epithelium.

**Figure 5:**
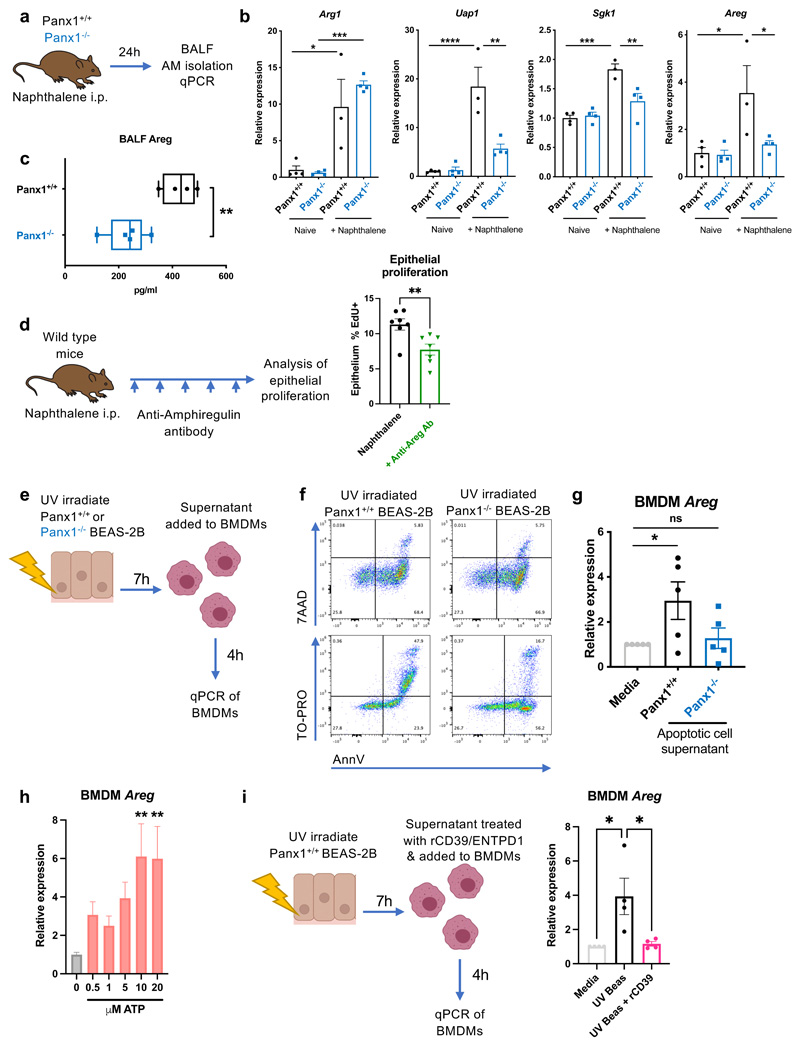
Dying cell pannexin1 imprints an injury phenotype upon macrophages. (a) Schema of experimental protocol for analysis of gene expression in lung alveolar macrophages at day 1 after naphthalene-induced epithelial injury in Panx1+^/^+ and Panx1^-/-^ mice. (b) qPCR analysis of *Arg1, Uap1, Sgk1 and Areg* in naïve and post-naphthalene injury alveolar macrophages from Panx1+^/^+ and Panx1^-/-^ mice (n-3-4/group, one experiment, assessed by one-way ANOVA with Tukey's multiple comparisons test). (c) Analysis of amphiregulin protein in BALF supernatant in Panx1+^/^+ and Panx1^-/-^ mice at day 1 after epithelial injury, analysed by Luminex (n=4-5/group, one experiment, assessed by t-test). (d) Schematic for neutralising amphiregulin *in vivo* by administration of anti-amphiregulin antibody (5μg in 200μL sterile PBS given intraperitoneally) on days 0,1,2,3,4,&4.5 after naphthalene injury, with epithelial proliferation assessed by EdU incorporation (n=7/group, two independent experiments, assessed by t-test). (e) Schema of experimental protocol for inducing apoptosis in Panx1+^/^+ or Panx1^-/-^ BEAS-2B epithelial cells with (f) confirmation of apoptosis induction (AnnV/7AAD staining) and Panx1 channel opening in Panx1+^/^+ apoptotic cells (AnnV/TO-PRO staining) and (g) apoptotic cell supernatant transferred onto mouse bone marrow derived macrophages (BMDMs) for 4h prior to qPCR for *Areg* (n=5, assessed by oneway ANOVA with Holm-Šídák's multiple comparisons test). (h) BMDMs were treated with increasing concentrations of the Panx1-released nucleotide ATP for 4h prior to qPCR for *Areg* (n=4, assessed by one-way ANOVA with Holm-Šídák's multiple comparisons test, each group compared to control/0μM ATP). (i) Degradation of extracellular ATP using recombinant CD39/ENTPD1 (rCD39; 44ng/ml) attenuates induction of *Areg* by BMDMs in response to apoptotic epithelial cell supernatants (qPCR after 4h; n-=4, assessed by one-way ANOVA with Holm-Šídák's multiple comparisons test). *p<0.05, **p<0.01. *** p<0.001, **** p<0.0001.

To test if Panx1 regulated macrophage phenotype post-injury, we collected bronchoalveolar lavage fluid (BALF) at 24h post naphthalene-induced injury and isolated Siglec-F+ alveolar macrophages (Fig. 5a). We performed qPCR on these macrophages to assess expression of genes that were recently shown to be induced by Panx1-dependent metabolites (14), namely *Uap1, Sgk1, and Areg* (which encode UDP-N-Acetylglucosamine Pyrophosphorylase 1, Serum/Glucocorticoid Regulated Kinase 1 and amphiregulin, respectively). Expression of these genes was induced in an injury- and Panx1-dependent manner, whereas expression of *Arg1* (Arginase 1; polyamine pathway) was regulated in an injury-dependent but Panx1-independent manner (Fig. 5b). *Areg*, a member of the epidermal growth factor (EGF) family of extracellular protein ligands, was of particular interest as it has documented roles in tissue repair, particularly in promoting epithelial proliferation and differentiation, and can be expressed by tissue macrophages (29). Furthermore, the EGF receptor itself promotes proliferation of airway epithelial cells after injury to facilitate repair (30). In support of this notion, when we assessed amphiregulin protein levels in BALF from Panx1^-/-^ mice after naphthalene injury, this was lower compared to control mice (Fig. 5c).

To test whether amphiregulin was functionally important in airway epithelial regeneration we targeted amphiregulin with the use of neutralizing anti-amphiregulin antibodies (Fig. 5d). Neutralizing amphiregulin led to attenuated injury-induced epithelial proliferation, confirming amphiregulin as functionally important in airway epithelial proliferation and repair (Fig. 5d). As a parallel in vitro approach to test whether Panx1 on dying cells regulates *Areg* induction in macrophages, we collected supernatants from apoptotic Panx1+^/^+ or Panx1^-/-^ BEAS-2B airway epithelial cells and added them to bone marrow-derived macrophages (Fig. 5e,f). This confirmed that soluble mediators from apoptotic cells can upregulate macrophage *Areg*, and that this *Areg* induction was attenuated when the apoptotic cells lacked Panx1 (Fig. 5g). We then tested whether ATP, a nucleotide released via Panx1 from apoptotic cells (17), was involved in *Areg* induction in macrophages. We also found that ATP treatment *in vitro* was sufficient to induce *Areg* in BMDMs (Fig. 5h). Furthermore, to test whether nucleotides within the supernatants of apoptotic epithelial cells were necessary for *Areg* induction, we treated the apoptotic supernatants with recombinant CD39/ENTPD1 to degrade extracellular ATP; when added to BMDMs, this led to diminished induction of *Areg* (Fig. 5i). Together, these experiments demonstrate that Panx1-released ATP is necessary (and ATP itself is sufficient) to induce macrophage amphiregulin in response to dying epithelial cells, and that amphiregulin functionally contributes to epithelial regeneration.

**Figure 6:**
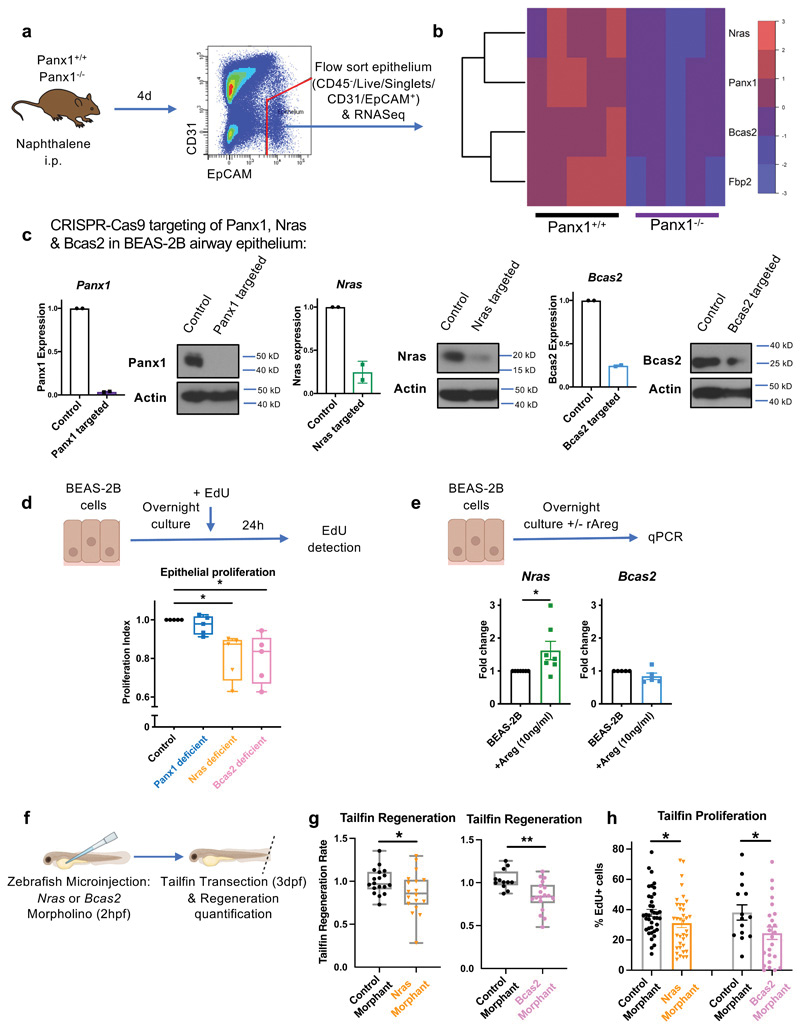
Pannexin 1 functions upstream of epithelial Nras and Bcas2 to drive injury-induced proliferation in the regenerating epithelium. (a) Schema of experimental protocol for RNAseq analysis of purified lung epithelium from Panx1^+/+^ and Panx1^-/-^ mice after naphthalene epithelial injury with (b) heat map of differentially regulated genes, units representing log2 scaled counts. (c) CRISPR–Cas9 genetic deletion of *Panx1, Nras* and *Bcas2* was performed in BEAS-2B epithelial cells (confirmation at transcript and protein level) with (d) proliferation of these cell lines measured by EdU incorporation over 24h, displayed relative to control (n=5, assessed by Kruskal-Wallis test with Dunn's multiple comparisons test). (e) Measurement of *Nras* (n=7) and *Bcas2* (n-5) in BEAS-2B cells after overnight treatment with recombinant human amphiregulin (rAreg) (assessed by Wilcoxon signed-rank test). (f,g) Generation of Nras and Bcas2 zebrafish morphants (or control sequence morphants) for assessment of regeneration rate at 48h post-tailfin transection (n=12-18 separate animals, two separate experiments, assessed by t-test) and (h) tailfin proliferation analysed by EdU incorporation (n=14-39 per group, assessed by t-test). *p<0.05, **p<0.01.

To better understand the potential mechanism by which Panx1 regulates epithelial proliferation and repair, we performed RNA sequencing on flow-sorted purified epithelium from Panx1+^/^+ and Panx1^-/-^ mice at day 4 after naphthalene-induced injury (Fig. 6a,b). This revealed four differentially regulated transcripts that were all reduced in epithelium collected from Panx1^-/-^ mice. These included: (1) *Panx1* itself (which served as an internal confirmation/ positive control); (2) *Fbp2* (Fructose-bisphosphatase 2) which catalyses the hydrolysis of fructose 1,6-bisphosphate, a metabolite that has been shown to be released via Panx1 from apoptotic cells (14); (3) *Nras* (encoding a version of the Ras GTPase); and (4) *Bcas2* (encoding the protein Breast Carcinoma Amplified Sequence 2). *Nras* and *Bcas2* were of particular interest as Ras family members are known to be activated downstream of EGF receptor signaling to regulate various physiological processes including proliferation (31), and *Bcas2* is an oncogene known to positively affect clonogenicity and migration of breast cancer cells (32, 33). Further, *Bcas2* is in the top 1% of host genes linked to SARS-CoV-2 infection per the 'metaanalysis by information content' (MAIC) algorithm (34), with severe disease characterized by impaired epithelial regeneration (5, 35).

We tested the functional role of these target genes identified in our RNAseq using BEAS-2B cells, a cell line derived from normal human airway epithelium. Cas9-GFP-expressing BEAS-2B cells were generated, and genetic deletion of *Panx1, Nras* or *Bcas2* was performed using CRISPR-Cas9. The fourth gene, *Fbp2* was not targeted as BEAS-2B do not express *Fbp2.* After confirmation of successful targeting of *Panx1, Nras,* and *Bcas2* (Fig. 6c), epithelial proliferation was assessed. Proliferation was intact in Panx1-deficient epithelium, but single deletion of either *Nras* or *Bcas2* led to reductions in cellular proliferation to around 80% of control levels (Fig. 6d). Given our previous data showing reductions in amphiregulin in response to tissue injury in Panx1^-/-^ mice, and the potential that either *Nras* or *Bcas2* could be functioning downstream of EGF receptor signalling, we investigated whether *Nras* or *Bcas2* could be directly upregulated by amphiregulin. Treatment of BEAS-2B cells with recombinant human amphiregulin led to an increase in *Nras;* in contrast, *Bcas2* levels were not directly altered by Areg (Fig. 6e). To further explore the role of *Nras* and *Bcas2* in epithelial regeneration *in vivo,* we performed genetic targeting in zebrafish prior to tailfin injury (Fig. 6f). This showed diminished tailfin regeneration (Fig. 6g) and diminished tailfin proliferation (Fig. 6h) in both *Nras* and *Bcas2* morphants. Taken together, these data suggest that Panx1 was not intrinsically driving epithelial proliferation but was establishing an injury-induced repair circuit that involves epithelial-macrophage bidirectional crosstalk, with *Nras* and *Bcas2* likely acting downstream of Panx1 to drive epithelial proliferation and tissue regeneration.

## Discussion

Epithelial structures are frequently damaged during challenges to organ homeostasis, with rapid repair being essential to restore organ function and protect the whole organism (36, 37). In the lung, the epithelium is simultaneously in intimate contact with both the external environment (air) and internal environments (blood, lymphatic vessels), making challenges to homeostasis frequent, with potential high-risk consequences to the individual after tissue injury. Indeed, acute pulmonary insults (bacterial pneumonia, viral infection, acute respiratory distress syndrome (ARDS)) and chronic lung diseases (e.g. asthma & COPD) are typified by epithelial damage and cell death (3). Nowhere has this been more acutely highlighted than the ongoing COVID-19 global health emergency, where overwhelming epithelial injury and aberrant repair contributes to respiratory failure and substantial mortality in severe cases (4, 5). Therefore, understanding mechanisms by which epithelial repair is achieved is essential to delineate novel targets that promote recovery after organ injury, especially within the lung where no existing therapies directly target lung regeneration. Dying cells are frequently generated during challenges to tissue homeostasis, but how these dying cells signal to the tissue microenvironment to drive efficient replacement of the cells lost by injury is not yet fully understood (1). Collectively, the data presented in this work identifies and provides key insights into several distinguishable steps in this process, and we advance a concept that Panx1 activation during organ injury and cell death imprints a repair program upon nearby macrophages that in turn promotes epithelial repair.

First, we show that the presence of Panx1, a cell membrane channel that is activated by caspases during cell death, drives efficient epithelial repair after tissue injury. While Panx1 has recently been shown to be important in controlling inflammation in diverse settings, including inflammatory arthritis and allergic airway inflammation, we hypothesized that Panx1 might also act as an important link between dying cells and tissue regeneration. By using acute injury and subsequent regeneration of the pulmonary airway as the major focus of our studies, we observed that absence of Panx1 led to a defect during the recovery phase. Parallel experiments in zebrafish, with inhibition or absence of Panx1, also demonstrated a requirement for Panx1 during tailfin regeneration after injury. This suggests that the role of Panx1 in tissue repair is conserved between species and it may therefore regulate tissue repair in response to diverse insults.

Second, as tissue macrophages are abundant in heathy tissues and have roles in diverse pathophysiological contexts (2), we explored macrophage function during airway injury and repair. We found that in response to airway injury, pulmonary macrophage numbers expand and that these macrophages are critical for epithelial regeneration. In human lung injury, macrophage expansion has been reported by several groups including our own (4, 5), with their roles and potential function in post-injury repair are rapidly being explored. How macrophages sense injured tissue and then mount an appropriate response remains largely unclear. Our data presented here demonstrate that Panx1-dependent communication between dying epithelial cells and macrophages can imprint an injury phenotype upon tissue macrophages, which in turn enhances macrophage production of epithelial mitogens such as amphiregulin. We find that upregulation of macrophage *Areg* in response to dying epithelial cells is primarily driven by epithelial Panx1 and likely occurs via Panx-1-released extracellular ATP. Amphiregulin is a well-recognised mitogen that can promote epithelial regeneration of tissues. In the context of *Nippostrongylus brasiliensis* infection in the lung, amphiregulin has been implicated (29), although the mechanism of ATP release *in vivo* was not explored. In this report, we demonstrate that neutralisation of amphiregulin during naphthalene-induced epithelial injury and regeneration led to a reduction (by a third) in epithelial proliferation, suggesting a critical and non-redundant contribution of amphiregulin in our system. This effect is similar to that observed in testing IL-22 in tracheal epithelial repair after influenza injury (38), 39). Panx1-mediated signals may be especially important within the structural hierarchy of a given organ where the dying cells may be anatomically segregated from nearby macrophages. Within the lung, this may be especially relevant, where alveolar macrophages are present within the luminal aspect of alveoli and small airways (adjacent to apical surface of epithelium) and interstitial macrophages are present within lung parenchyma. Soluble signalling, dependent on Panx1 opening after epithelial cell injury, may therefore allow rapid signalling of disrupted and injured tissue to disparate populations of cells, allowing for maximal responses and efficiency in tissue regeneration. The exact roles played by alveolar vs. interstitial macrophages during airway injury, and the potential for crosstalk between these two cell types, remains to be fully explored. Whether each macrophage population performs a distinct function, or whether depletion of a single population leads to a reduction in 'global' lung macrophage numbers, will require further study. Similarly, while we have focused on Panx1-mediated signalling to macrophages during tissue injury, Panx1 likely permit injury signals to reach cells of other lineages including fibroblasts and endothelium, enabling them to synchronously mount an appropriate response to injury.

Third, we find that epithelial injury-induced proliferation is boosted downstream of both Panx1 and macrophages, with proliferation of epithelium required to replace cells lost during injury. Switching from a quiescent state with extremely low levels of basal proliferation to rapid and coordinated epithelial proliferation clearly requires substantial changes to the local microenvironment. Through RNASeq analysis we found that Panx1 indirectly boosts epithelial injury-induced proliferation, with Panx1 promoting expression of *Nras* and *Bcas2* in the regenerating epithelium. Furthermore, we found that *Nras* and *Bcas2* both positively regulate epithelial proliferation and regeneration *in vivo.* Interestingly, *Nras* is widely expressed, and both *Nras* and *Bcas2* have also been implicated in growth of epithelial tissue during carcinogenesis (32, 40), and Panx1 expression is associated with adverse outcomes in lung, renal and endometrial cancer (The Human Protein Atlas). This suggests that these molecules may regulate epithelial growth in diverse settings, including those considered to be beneficial (e.g. organ regeneration after injury) and detrimental (e.g. carcinogenesis). Furthermore, *Bcas2* is in the top 1% of host genes implicated in SARS-CoV-2 infection based on the meta-analysis by information content (MAIC) algorithm (34), with severe disease characterized by widespread lung epithelial injury and impaired regeneration (5). Future studies are needed to explore whether molecules such as amphiregulin may directly contribute to *Nras* and *Bcas2* expression or function. While we focused on Panx1-dependent induction of the mitogen amphiregulin within macrophages, we do not rule out other Panx1-dependent factors playing a role in tissue regeneration.

The factors and molecules that macrophages use to survey their local microenvironment to detect challenges to organ homeostasis and how they respond to promote tissue repair and re-establish homeostasis are of high relevance to both human and animal health. We propose that the Panx1-mediated bidirectional signalling pathway between epithelium and macrophages during tissue injury and regeneration has two essential components: first, Panx1 signals tissue injury and imprints an injury phenotype upon surrounding cells (for example via Panx1-released ATP signalling to macrophages); second, macrophages enhance tissue regeneration by boosting and coordinating injury-induced epithelial proliferation (including macrophage-derived amphiregulin signalling to the regenerating epithelium; Fig. S5). Future approaches to target this regeneration circuit may provide benefits for diseases associated with impaired tissue regeneration such as that caused by major organ injury.

## Materials and Methods

### Study design

The central question addressed in this work was on how the lung epithelium and macrophages communicate and interact to coordinate lung tissue regeneration after injury. Specifically, we tested the involvement of pannexin 1 (Panx1) channels that facilitate inter-cellular communication in other contexts. In our studies, airway epithelial injury was induced via naphthalene in mice lacking Panx1 either globally or in specific cell types. The response of epithelial cells as part of the tissue regeneration was assessed, including epithelial cell death after injury, proliferation as part of regeneration, and transcriptomics studies. The requirement and role of lung macrophage populations in regeneration was assessed by depleting specific lung macrophage populations. To test the involvement of Panx1 in communication between epithelial cells and macrophages, Panx1 was genetically deleted in macrophage cell populations, and by restoring Panx1 function via transgenic reexpression of Panx1 in epithelial cells. We also assessed the factors released via Panx1 from epithelial cells involved in the epithelial cell-macrophage communication, and tested the role of ATP and the induction and release from macrophages of amphiregulin, a soluble mitogen that promotes epithelial cell proliferation. We also tested the conservation of this Panx1-dependent epithelial: macrophage communication using genetic and pharmacological approaches in the regenerating zebrafish tailfin.

### Mice and In vivo model of airway epithelial injury

C57BL/6 wild type mice were ordered from Jackson or bred in house, with Cx3cr1-Cre (Stock No: 025524), LysM-Cre (Stock No: 004781) and YFP-reporter (Rosa26^STOP-EYFP^; Stock No: 006148) mice from Jackson. Panx1^fl/fl^, Panx1 global KO mice (Panx1^-/-^) and Panx1-transgenic (Panx1^Tg^) mice have previously been described (14, 28). All mice were bred and maintained in specific pathogen-free conditions. For in vivo experiments female mice aged 8-14 weeks were used, with all experiments approved by the Institutional Animal Care and Use Committee (IACUC) at the University of Virginia, or performed in accordance with the UK Home Office Animals (Scientific Procedures) Act of 1996 after review by local ethics committee. Naphthalene was dissolved in sterile corn oil and administered as a single i.p. injection at a dose of either 160mg/kg or 200mg/kg as per figure legends. Analysis of lung injury and immune cell infiltration was achieved by surgical ligation of the left hilum prior to perfusing the right lobes with 5ml sterile PBS via the right ventricle. The left main bronchus was then cannulated and the left lobe inflated with formalin prior to paraffin embedding and H&E staining or IHC on sections. Perfused right lobes and trachea were excised and cut into small pieces with scissors before digestion in 2mg/ml type II collagenase (Worthington) in the presence of Ca^2+^/Mg^2+^. Lung cells were further disaggregated by passing through a 18G then 20G needle, prior to passing the cell suspension through a 40μm filter. Cells were washed in RPMI and spun at 300g before RBC lysis in Tris-buffered ammonium chloride buffer, and then resuspended in PBS with 0.2% BSA for flow cytometry. For measurement of proliferation mice received 1.5mg of EdU (5-ethynyl-2'-deoxyuridine) in 200uL sterile PBS at 24h and 12h prior to tissue collection. In separate experiments bronchoalveolar lavage (BAL) was acquired via delivery of 1.0 ml of cold PBS intratracheally through a canula. BAL fluid was centrifuged at 300g, and supernatants were frozen at - 80°C for subsequent cytokine analysis via Luminex or ELISA, with pelleted BAL cells further analysed by qPCR or flow cytometry. H&E stained lung sections were analysed by a thoracic pathologist blinded to the experimental group and each field of view assigned a score of 1-Normal appearance/confluent epithelium, 2-Mild loss of confluence, 3-Significant loss of nuclear confluence & disorganization 4-Significant loss of nuclear confluence & irregular/haphazard nuclear arrangement, with a repair score of 1/average score given. Cleaved caspase-3 staining (Cell Signaling 9661) was performed with automated cell analysis by QuPath software on digitized images. TAT-Cre (Merck) was administered intratracheally (i.t.) (50units/50μL).

### Zebrafish model of sterile epithelial injury

Sterile tailfin transection of the median tailfin was performed on 3 days post fertilization (3dpf) embryos as previously described with each embryo dispensed in a single well of a 48 well plate and imaged as per figure legends (10). Tailfin regeneration rate was calculated as the increase in tailfin area from 3dpf to 5dpf, normalised to the increase in the control group. Microinjection of *panx1a* morpholino (5'-CATATGTTGTATGCGCTTGCCTTAT-3'; 1nL 1mM, Gene Tools) or a non-targeting control (5'- CCTCCTACCTCAGTTACAATTTATA-3') at the 1-4 cell stage was carried out as previously described (21). Microinjection of *nras* (5'- AACAACCAGCTTATACTCAGTCATC-3') 0.5mM or *bcas2* (5'- GCTGGTCCTGCCATCCTGAACAAAC-3') 50μM morpholino (or respective non-targeting control) was similarly performed. Panx1 inhibitors Trovafloxacin (80mM) and Spironolactone (120mM) were dissolved in sterile DMSO and 1nL microinjected into the yolksac prior to tailfin transection as previously described (10). For analysis of proliferation in the regenerating tailfin, zebrafish were bathed in 1mM EdU at 24-48 hpi prior to fixation in 4% PFA and staining with Click-iT™ EdU Cell Proliferation Kit for Imaging, Alexa Fluor™ 594 dye. EdU incorporation was assessed by light sheet fluorescent microscopy (LSFM) and semi-automated quantification in FIJI using the Trackmate plugin (41). To generate macrophage deficient zebrafish, the Tg(csf1ra:gal4;UAS:mCherry-NfsB) zebrafish line was utilized, which expresses nitroreductase enzyme (NfsB) under the control of the macrophage promotor csf1ra (fms) (42). Bathing of larvae in 0.5mM metronidazole in 0.2 % DMSO at 2-3 dpf ablated macrophages prior to tail fin transection.

### Lung macrophage or amphiregulin manipulation

Alveolar macrophages were depleted by clodronate liposomes administered intratracheally (100μl) 24h prior to experimentation. Interstitial macrophages were depleted by the CSF1r inhibitor PLX5622, a gift from Plexxicon; animals were fed PLX5622 containing chow (1200mg/kg feed) ad libitum for 3d prior to experimentation, with additional PLX5622 (65mg/kg) given by oral gavage (on days 0,1,2,3&4 of naphthalene administration to counteract illness associated anorexia). Amphiregulin was targeted in vivo by a neutralizing antibody (AF989, R&D), with 5μg in 200μl sterile PBS administered i.p. on days 0, 1, 2, 3, 4 and 4.5 after naphthalene administration.

### Flow cytometry quantification of immune cells and epithelial proliferation

A fixed volume of total lung cells after collagenase digest were stained for major immune cell populations in the presence of Fc block (anti CD16/CD32, eBioscience clone 93) and LIVE/DEAD™ Fixable Yellow Dead Cell Stain at 4°C for 30 mins, using combinations of anti-CD45 (eBioscience clone 30-F11), CD11b (Biolegend clone M1/70), CD11c (Biolegend clone N418), MHCII (Biolegend clone M5/114.15.2), CD24 (BD Biosciences clone M1/69), Siglec-F (BD Biosciences clone E50-2440), Ly6G (Biolegend clone 1A8), CD64 (Biolegend clone X54-5/7.1) (Myeloid cells); anti-CD45, CD11b, CD11c, MHCII, CD64, F4/80 (eBioscience clone BM8), MerTK (eBioscience clone DS5MMER) (macrophage); anti-CD45, EpCAM (eBioscience G8.8), CD31 (eBioscience clone 390) (Epithelial cells). Cells were then washed and resuspended in PBS with 0.2% BSA and a fixed volume run on an Attune flow cytometer to allow quantification of immune cells. EdU was detected using a Click-it EdU imaging kit (Fisher) as per manufacturer's instructions after fixation in 10% formalin (15 mins) and permeabilization in 0.07% saponin, with additional azide from Click Chemistry Tools.

### RNA Sequencing

After induction of naphthalene induced injury, lung epithelium was purified by cell sorting (CD45^-^/CD31^-^/EpCAM^+^ live singlets) and RNA isolated by NucleoSpin RNA isolation kit with on-column DNase digestion (Machery-Nagel). mRNA libraries were prepared using the Truseq Stranded mRNA kit following the manufacturers protocol. Libraries were then sequenced using an Illumina NextSeq 500 sequencer at 75 bp, paired-end reads with approximately 20 million reads per sample. Five independent replicates were sequenced for epithelium from Panx1+^/^+ and Panx1^-/-^ mice. Reads were checked for quality using FASTQC (v0.11.8), trimmed using BBMAP (v3.8.16b), and aligned to the mouse genome with GENCODE (vM22) annotations using STAR (v2.7.1a). Transcripts per million calculations were performed by RSEM (v1.3.1), the results of which were imported into R (v4.0.2) and Bioconductor (v3.12) using tximport (v1.18.0). Significant genes were called using DESeq2, using fold change cutoffs and pvalue cutoffs of 0.5 and 0.05 respectively. Results were visualized using Heatplus (v2.36.0), PCAtools (v2.2.0), and UpSetR (v1.4.0). Data code available on request.

### Cell Culture, in vitro proliferation measurement and apoptosis induction

BEAS-2B cells were cultured on collagen coated TC flasks in RPMI containing 2% serum. Cells were plated in 24 well plates at a density of 5x10^4^ cells per well in serum free RPMI overnight prior to addition of 10 μM EdU. Proliferation was measured as EdU incorporation after 24 hours measured by flow cytometry. In separate experiments BEAS-2B cells were incubated with 10ng/ml recombinant amphiregulin (rAreg) overnight prior to qPCR. For induction of apoptosis, BEAS-2B were maintained at sub-confluent density, trypsinised and resuspended in RMPI +0.2%FBS + 10uM glycine at a density of 2x10^6/ml and irradiated with 150mJ/cm^2^ using a CL-1000 UV Crosslinker. Apoptosis was confirmed after 7 hours by Annexin V (AnnV) and 7-AAD staining, with Panx1 opening confirmed with TO-PRO staining along with AnnV (43). Supernatants from the apoptotic epithelial cells were centrifuged at 300g for 5mins to pellet cells and then 4,000g for 20mins to pellet smaller particles prior to treating 5-7 day bone marrow macrophages grown in the presence of 50ng/ml rCSF-1 (BioLegend) for 4h. Degradation of extracellular ATP from the apoptotic cell supernatants was achieved by addition of recombinant CD39/ENTPD1 (rCD39, R&D), at a final concentration of 44ng/ml.

### CRISPR-Cas9 deletion

Cas9 and GFP expressing BEAS-2B airway epithelial cells (BEAS-2B from ATCC) were generated by lentiviral transduction using lentiCas9-EGFP plasmids and flow sorting of GFP+ cells. Cas9 expression was confirmed by immunoblotting using anti-Cas9 antibody (Biolegend, Cat. no: 844301). The following guide RNA sequences were designed and cloned to lentiGuide-Puro plasmid to generate knock out cells: Panx1, AGTCTGGAAACCTCCCACTG; Nras, AATGACTGAGTACAAACTGG; Bcas2, TTCAAAGGCAGAATAATCCG. Scrambled guide RNA, GTATTACTGATATTGGTGGG, was used as a control.

### PCR and immunoblotting

RNA was extracted using the RNeasy Mini Kit (Qiagen) or NucleoSpin RNA isolation kit (Machery-Nagel) and cDNA synthesized using the QuantiTect Reverse Transcription Kit (Qiagen) as per manufacturer's instructions. Taqman probes (Fisher) were used to measure gene expression using a StepOnePlus Real Time PCR System (Applied Biosystems). For immunoblotting tissue or cells were lysed in RIPA buffer with protease inhibitors and probed with anti-Pannexin1 (Cell Signalling 91137S) anti-Nras (Biolegend 866101) or anti-Bcas2 (Insight Biotech sc-365346) prior to analysis with chemiluminescence.

### Software

Flow cytometry was analysed by Flowjo (v10) with graphical representation of data using GraphPad Prism (v7 and v9). Additional images were generated using BioRender.com.

### Statistical analysis

Statistical analysis was performed using GraphPad Prism (v7 and v9), with the statistical test used in each experiment detailed in the figure legends, and N values referring to biological replicates.

## Supplementary Material

Supplementary Materials

## Data Availability

RNA sequencing data has been deposited in GEO under accession number GSE181101. All other data needed to support the conclusions of the paper are present in the paper or Supplementary Materials.

## One sentence summary

Epithelial-macrophage communication via pannexin channels promotes epithelial cell proliferation and repair after tissue injury
