# Supplementary material for: Pannexin 1 drives efficient epithelial repair after tissue injury
Source: Sci Immunol. Author manuscript; Available in PMC 2022 May 26. (PMC7612772; doi:10.1126/sciimmunol.abm4032)

Supplementary Materials:

Fig. S1. Acquisition and gating strategy for analysis of BALF cells post naphthalene.

Fig. S2. Analysis of lung myeloid cells.

Fig. S3. Depletion of alveolar and interstitial macrophage populations.

Fig. S4. Cre expression within alveolar and interstitial macrophage populations using Cx3Cr1-Cre mice.

Fig. S5. Graphical abstract of Pannexin1-regulated epithelial repair.

Data file S1. Raw data file (Excel file).

Figure S1. Acquisition and gating strategy for analysis of BALF cells post naphthalene

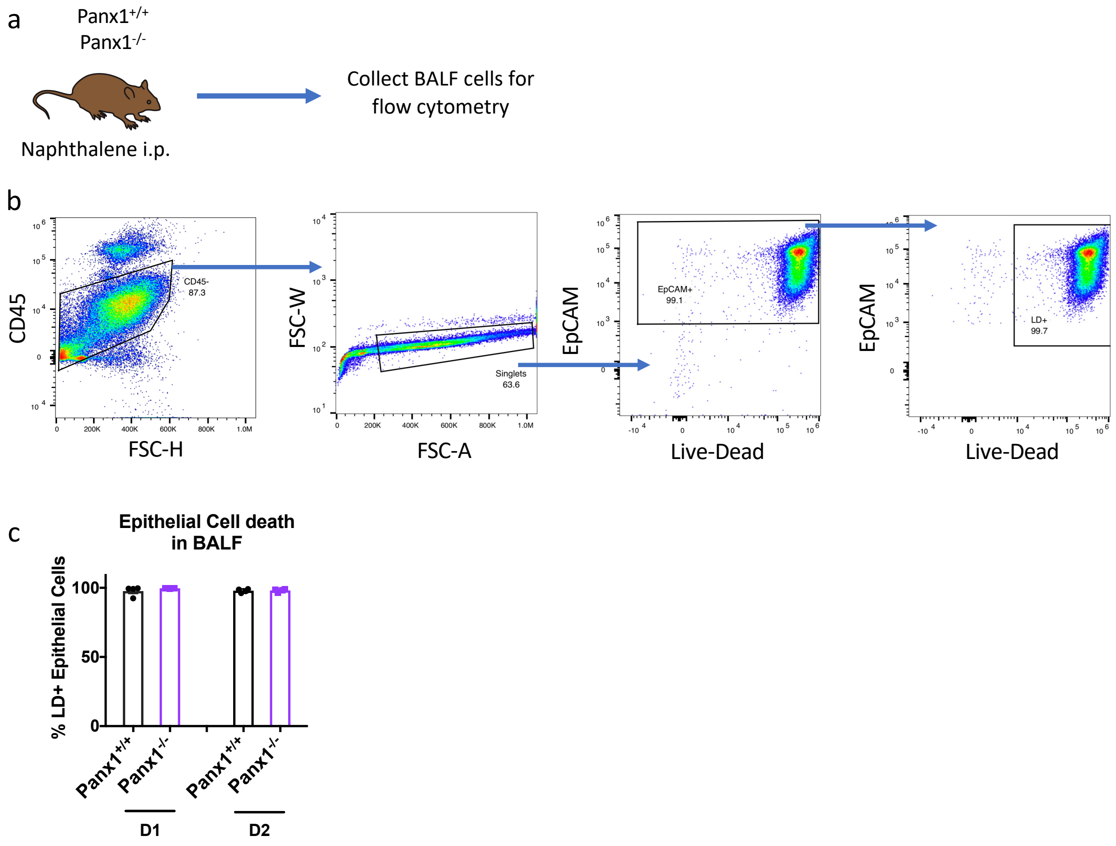

Figure S1. Acquisition and gating strategy for analysis of BALF cells post Naphthalene. (a) Experimental schema and (b) gating strategy for analysis of epithelial cells ($CD45^-$/$EpCAM^+$ cells) and their staining with a live-dead marker (c) at day 1 and 2 after naphthalene-induced epithelial injury (n=4-5).

Figure S2. Analysis of lung myeloid cells.

a

CD45⁺ live singlets:

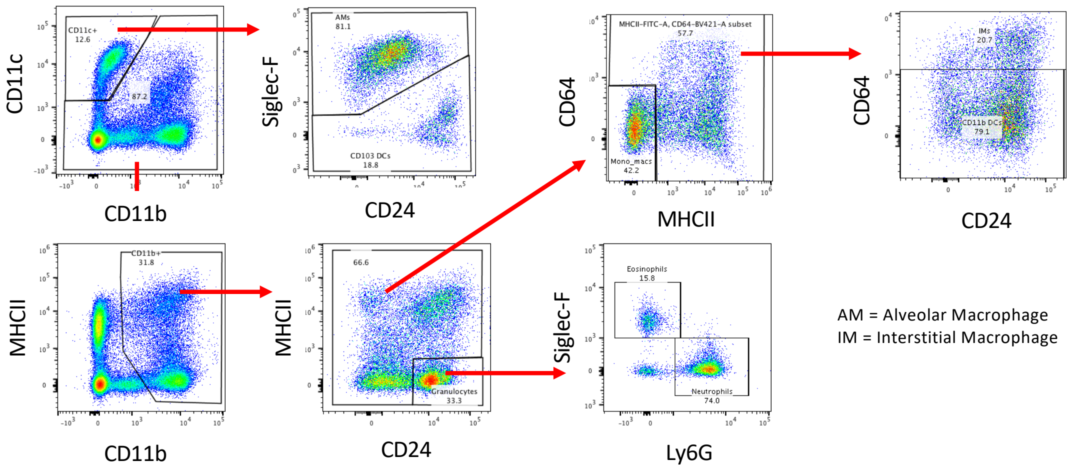

Figure S2. Analysis of lung myeloid cells. (a) Gating strategy to identify major myeloid cell subsets in digested lung tissue.

Figure S3. Depletion of alveolar and interstitial macrophage populations.

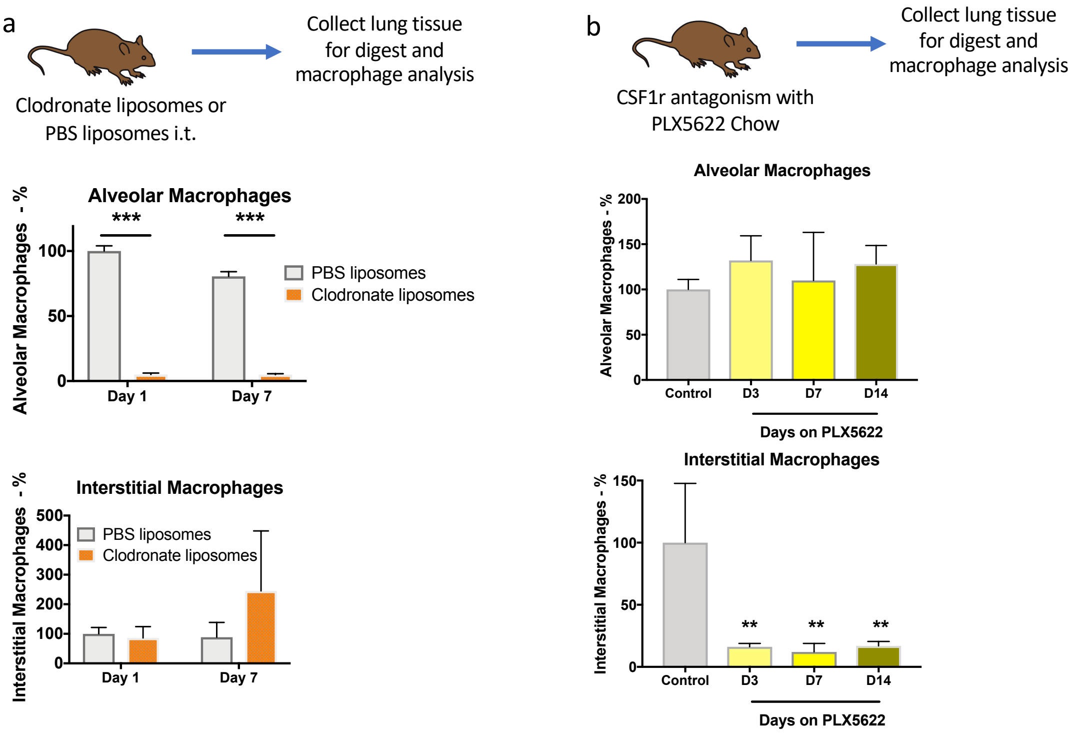

Figure S3. Depletion of alveolar and interstitial macrophage populations. Strategy to deplete (a) Alveolar macrophages by liposomal clodronate or (b) interstitial macrophages by the CSF1r antagonist PLX5622 from lungs of naïve mice. (n=3-4/group), **p<0.01, *** p<0.001

Figure S4. Cre expression within alveolar and interstitial macrophage populations using Cx3Cr1-Cre mice.

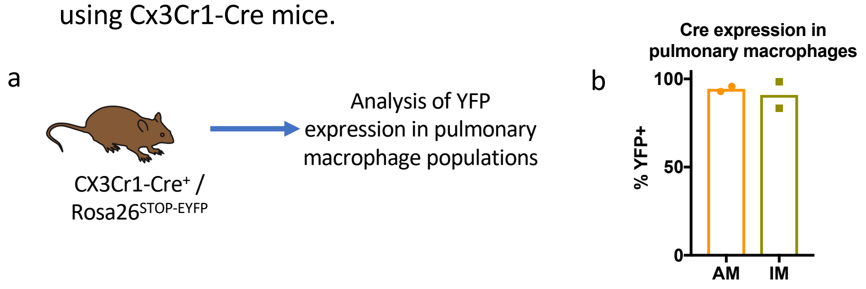

Figure S4. Assessing Cre expression within lung alveolar and interstitial macrophage populations using Cx3Cr1-Cre and YFP reporter mice. (a) Experimental schema and (b) the percentage of alveolar macrophages (AM) and interstitial macrophages (IM) expressing Cre by YFP.

Figure S5. Graphical abstract of Pannexin1-Regulated Epithelial repair

a

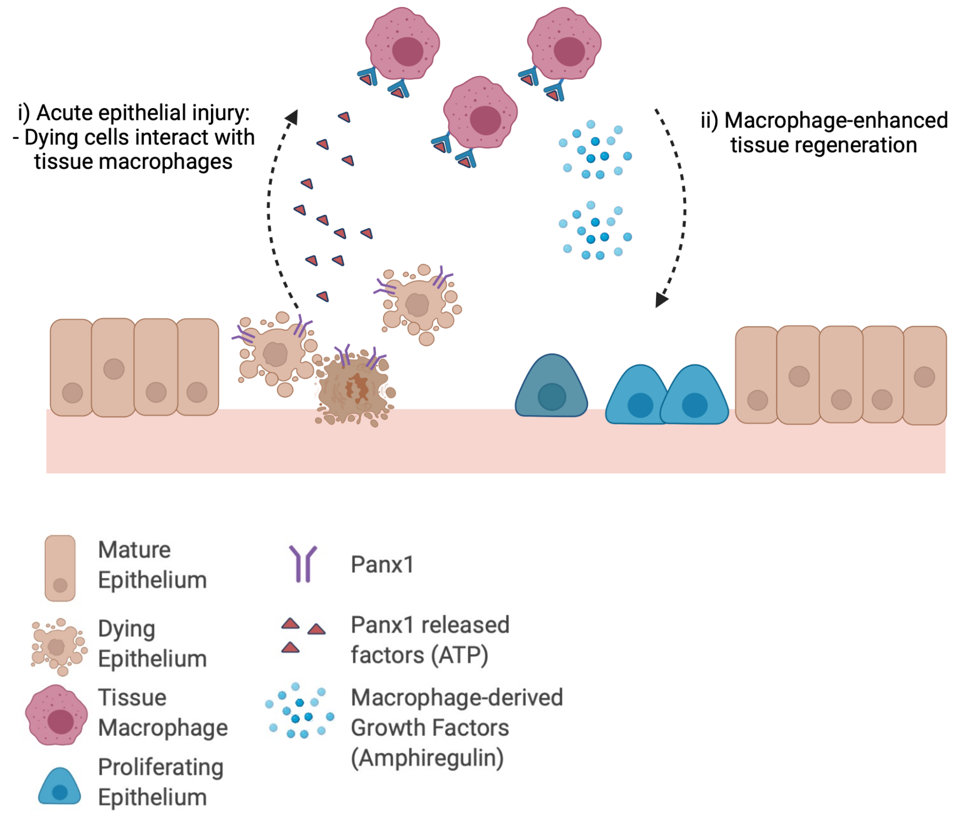

Supplement: Supplementary Materials [file EMS145048-supplement-Supplementary_Materials.pdf]
